# Advances in *Yarrowia* Genus Exploitation: From Fundamental Research to Industrial Biotechnology

**DOI:** 10.3390/foods14203502

**Published:** 2025-10-14

**Authors:** Joanna Kobus, Katarzyna Wierzchowska, Aleksandra Piotrowicz, Agata Urszula Fabiszewska

**Affiliations:** Department of Chemistry, Institute of Food Sciences, Warsaw University of Life Sciences-SGGW, Nowoursynowska 159c, 02-776 Warsaw, Poland; katarzyna_wierzchowska@sggw.edu.pl (K.W.); aleksandra_piotrowicz@sggw.edu.pl (A.P.); agata_fabiszewska@sggw.edu.pl (A.U.F.)

**Keywords:** biotechnology, food technology, unconventional yeasts, *Yarrowia*

## Abstract

Unconventional yeasts, most notably those belonging to the genus *Yarrowia*, are garnering mounting interest from the scientific community due to their considerable promise in biotechnological applications. In the scientific literature, most attention is devoted to the species *Y. lipolytica*. The present work focuses on presenting the detailed phenotypic and metabolic characteristics of other less studied species, such as *Y. bubula, Y. deformans, Y. phangngensis,* and *Y. alimentaria*. The review includes a comprehensive analysis of *Yarrowia* species, focusing on their taxonomy, ecology, physiology, and industrial potential. These yeasts demonstrate significant variability in terms of temperature tolerance, substrate utilization, enzymatic activity, and lipid accumulation. A comparative analysis of strain collections, genomic features, and published biochemical studies is conducted. Several described species possess characteristics that are suitable for many applications, including protease production, adaptation to low temperatures, and synthesis of valuable lipids and sugar alcohols. This review also presents a regulatory framework supporting the safe use of *Yarrowia* yeast species in food, feed, and pharmaceuticals, and discusses the genetic background of those microorganisms. Although *Y. lipolytica* is the most prevalent species in current applications, the growing knowledge of other *Yarrowia* species suggests significant untapped potential. It is imperative that further comparative, safety application, and genomic studies be conducted in order to fully exploit this diversity for the purpose of sustainable biotechnological innovation.

## 1. Introduction

In recent years, there has been a notable increase in the interest surrounding unconventional yeasts, largely due to their substantial potential in the domain of biotechnology. In the genus under consideration, *Yarrowia* occupies a special place, with *Yarrowia lipolytica* being its most recognizable and most frequently studied representative. The biomass of these yeasts is a valuable source of bioactive and nutritional compounds, including high protein content with a useful amino acid composition and high storage lipids with beneficial fatty acids [1]. Moreover, the yeast cells contain valuable micronutrients, including chromium and selenium in organic forms, along with vitamins and minerals, which increase their nutritional and biotechnological value. It is also worth mentioning that *Yarrowia* yeast is a rich source of fibre, with an approximate content of 25% [2]. This review aims to deepen our knowledge about species other than *Y. lipolytica*, lesser-known species of the genus *Yarrowia*, as well as to compare their unique characteristics, biotechnological potential, and the current state of research in this field. There are many papers describing *Y. lipolytica* and dedicated handbooks about biotechnological applications [3], genetics, genomics, and physiology [4]. Well-known researcher teams in these fields are led by the following: Catherine Madzak, who focuses on metabolic engineering of *Y. lipolytica* [5,6]; Waldemar Rymowicz, who studies the production of erythritol and citric acid by *Y. lipolytica* [7,8]; Svetlana Kamzolova, who works on biotechnological applications in *Y. lipolytica*, and also on acid production [9]; and Anita Rywińska, who investigates citric acid and erythritol production [10]. Also, the team led by Patric Fickers and the team by Marc Nicaud, who work on *Yarrowia* lipase characteristics and applications, followed by Seraphim Papanikolaou and George Aggelis’ team, who focus on single-cell oil biosynthesis [11,12]. Additionally, several well-known international groups have made significant advances in metabolic engineering and synthetic biology of *Y. lipolytica,* including Hal S. Alpers’ team, which specializes in metabolic engineering of *Y. lipolytica* [13,14]; and Hairong Cheng’s team, which focuses on the production of limonene, erythritol, D-mannitol, β-carotene, and sugar biosynthesis in *Y. lipolytica* [15,16,17]. These references are provided as examples of their work. Further information on *Y. lipolytica* can be found in the works of the above-mentioned authors. This article provides the most important information that allows for *Y. lipolytica* to be distinguished from other species and compared with them.

## 2. The *Yarrowia* Genus: General Characteristics and Research Developments

The first species of the *Yarrowia* yeast was identified by David Yarrow in 1980, who is responsible for the identification of the yeast genus, distinguishing it by the characteristic shape of the ascospores and the presence of coenzyme Q-9. The generic name *Yarrowia* was introduced in 1980 by mycologists van der Walt and von Arx [18]. The species name of *Yarrowia* yeast may derive from the place of discovery or the function it performs. The following table shows the taxonomy of the *Yarrowia* species (Table 1).

**Figure 1:**
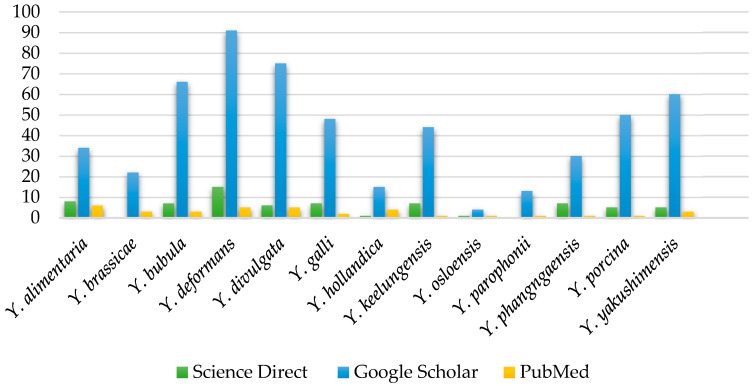
Number of publications about *Yarrowia* species from Science Direct, Google Scholar, and PubMed, excluding *Y. lipolytica* species [data collected and analyzed; accessed 6 June 2025 and 1 October 2025].

The genus *Yarrowia* currently includes 13 formally described species. To assess the level of scientific interest in each taxon, the number of publications indexed in the ScienceDirect, Google Scholar, and PubMed databases was analyzed [19,20,21]. The selection of these two databases was made on the basis of their comprehensive coverage of the peer-reviewed literature and accessibility; ScienceDirect provides a curated collection of high-quality journal articles, whereas Google Scholar captures a broader spectrum of scholarly outputs, including conference proceedings and preprints. Whilst it is to be expected that there will be some overlap in the publications retrieved from the two sources, employing both of them enables a more comprehensive overview of the research landscape to be obtained. The results are presented in Figure 1.

Figure 1 shows the distribution of publication counts for individual *Yarrowia* species. The clear research focus remains on *Y. lipolytica*, which is not included in the figure due to its significantly higher number of publications—approximately 7600 articles in ScienceDirect, 29,100 results in Google Scholar (as of 6 June 2025), and 2793 results in PubMed (as of 1 October 2025). In contrast, the remaining species are relatively underexplored. Among them, *Y. deformans, Y. divulgata, Y. bubula*, and *Y. galli* appear most frequently, with the majority of records retrieved from Google Scholar.

**Figure 2:**
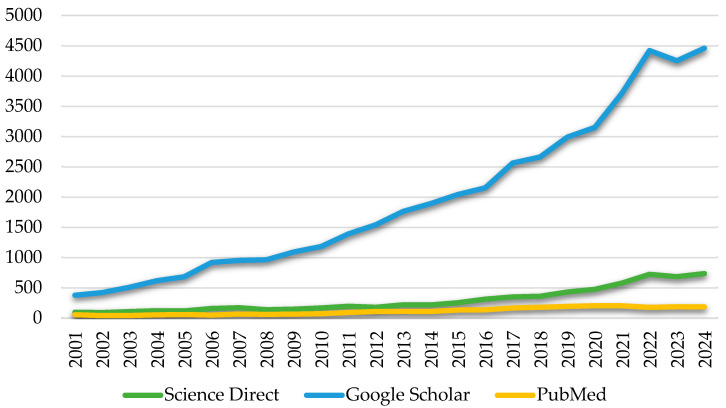
Scientific publications on *Yarrowia* between 2001 and 2024: a comparison of ScienceDirect, Google Scholar, and PubMed [data collected and analyzed; accessed 6 June 2025 and 1 October 2025].

Figure 2 presents the growth in the number of publications on the genus *Yarrowia* (including *Y. lipolytica*) between 2001 and 2024. The data demonstrates a consistent increase in research activity, with marked acceleration after 2015. This trend likely reflects the expanding use of *Yarrowia* species in industrial biotechnology. While the bibliometric analysis highlights the dominant role of *Y. lipolytica* in the current scientific literature (Figure 2), other species of the genus have also been isolated and described in recent years, although they remain relatively understudied.

The distribution of known *Yarrowia* species across global microbial collections and isolation environments is summarized in Table 2. A significant majority of deposited strains belong to *Y. lipolytica*, with over 230 entries distributed among several major international culture repositories, including CBS—Westerdijk Fungal Biodiversity Institute in the Netherlands; KKP—Polish Collection of Industrial Microorganisms; MUCL—The Belgian Coordinated Collections of Microorganisms; CLIB—CIRM-Levures Yeast Collection in France; Y.—National Collection of Agricultural and Industrial Microorganisms in Hungary; and C-—VTT Culture Collection in Finland. This underscores *Y. lipolytica*’s status as the most extensively studied and utilized species within the genus, reflecting its broad application in industrial biotechnology and synthetic biology.

Strains of *Y. lipolytica* have been isolated from a wide geographical range, encompassing Europe, Asia, North and South America, and even the Southern Ocean (Table 2). The environmental diversity is equally broad, with sources including dairy products, soil, food industry environments, aquatic ecosystems, petrochemical sites, and the human body. This suggests an exceptional level of ecological plasticity and adaptation, consistent with its metabolic versatility. In contrast, other *Yarrowia* species are represented by fewer strains, typically confined to one or two countries and narrow ecological niches. Some exhibit apparent host or habitat specificity—for instance, *Y. parophonii* has only been isolated from the gut of the carabid beetle *Paraphonus hirsutulus* in Bulgaria, while *Y. keelungensis* was recovered from seawater in Taiwan, and *Y. yakushimensis* from the gut of a Japanese termite. These examples point to possible ecological specialization or the undersampling of diverse habitats.

Table 2 presents a collection of the most popular *Yarrowia* yeast collections. A collection of these and other strains found in Europe and around the world can also be found on the GCM (Global Catalogue of Microorganisms) [22] and ECCO (European Culture Collections’ Organisation) [23], which list all of the institutions holding individual strains.

**Table 2:** Overview of currently recognized species of the genus *Yarrowia*.

Species	Number of Strains *	Region of Isolation	Isolation Source	Reference
*Yarrowia lipolytica*	42 (CBS) 6 (KKP) 31 (MUCL) 6 (C-) 124 (CLIB) 21 (Y.), 16 (ACA-DC)	Netherlands, Turkey, Southern Ocean, China, Norway, Germany, Russia, United Kingdom, United States of America, France, Argentina, Italy, Poland, Morocco, Belgium, Portugal, Finland, Spain, Czech Republic	human, dairy products, water and aquatic environments, soil, natural environment, food and food fermentation, food industry processes, industrial and petrochemical, unspecified,	[24,25,26,27,28,29,30]
*Yarrowia alimentaria*	3 (CBS) 1 (C-) 3 (Y.)	Norway, Finland, Hungary	Brie Régalou cheese; cured ham; yoghurt production line; fast food, minced beef and pork	[24,28,30]
*Yarrowia brassicae*	1 (CBS)	China	pickled cabbage	[24]
*Yarrowia bubula*	1 (CBS) 5 (Y.)	Hungary	beef and pork	[24,28]
*Yarrowia deformans*	18 (CBS) 1 (KKP) 5 (MUCL) 4 (CLIB) 1 (Y.), 1 (ACA-DC)	Belgium, France, South Africa, Japan, Germany, Austria, Poland, Hungary	frozen chicken; pyrenees cheese; moss; soil; lichens; fish conserves; human; fermented beverage; tick	[24,25,26,27,28,31]
*Yarrowia divulgata*	1 (CBS) 4 (Y.)	Denmark, United States, Hungary	bacon, chicken liver and breast, gryphon, minced beef	[24,28]
*Yarrowia galli*	2 (CBS) 1(C-) 8 (Y.)	United States of America, Finland	chicken liver and breast, pears	[24,28,30]
*Yarrowia hollandica*	2 (CBS)	Belgium, Netherlands	Caprice Des Dieux cheese; back of a cow	[24]
*Yarrowia keelungensis*	1 (CBS)	Taiwan	seawater	[24]
*Yarrowia osloensis*	4 (CBS)	Norway	yoghurt with melon; yoghurt with coconut; yoghurt with kiwi	[24]
*Yarrowia parophonii*	7 (CBS)	Bulgaria	gut of *Paraphonus hirsutulus* (carabidae)	[24]
*Yarrowia phangngaensis*	1 (CBS)	Thailand	seawater	[24]
*Yarrowia porcina*	2 (CBS) 6 (Y.)	Hungary, Brazil	minced beef and pork; subsurface water of the Rio Doce river	[24,28]
*Yarrowia yakushimensis*	4 (CBS) 4 (Y.)	Japan	gut of Japanese termite (hodotermopsis sjoestedti)	[24,28]

* Strain collection abbreviations. CBS—Westerdijk Fungal Biodiversity Institute, Netherlands. KKP—Polish Collection of Industrial Microorganisms, Poland. MUCL—The Belgian Coordinated Collections of Microorganisms, Belgium. CLIB—CIRM-Levures Yeast collection catalog, France. Y.—National Collection of Agricultural and Industrial Microorganisms, Hungary. ACA-DC—Agricultural College of Athens-Dairy Collection, Greece. C- —VTT, Culture Collection, Finland.

Several species, such as *Y. deformans* and *Y. alimentaria*, exhibit broader ecological distributions. *Y. deformans*, in particular, has been recovered from diverse environments including cheese, frozen poultry, soil, lichen, moss, fermented beverages, and even arthropods like ticks—suggesting high adaptability and potential for both environmental and applied significance. Moreover, multiple *Yarrowia* species have been isolated from fermented foods and dairy environments, such as *Y. osloensis* from flavoured yoghurts or *Y. hollandica* from cheese and cattle skin. These findings suggest that *Yarrowia* yeasts may be more common in food-related ecosystems than previously assumed and could represent a reservoir of useful traits for food biotechnology and microbial fermentation.

Altogether, this ecological and geographic mapping highlights the genus *Yarrowia* as an ecologically diverse group of yeasts. While *Y. lipolytica* remains the best-characterized species, a growing number of isolates from lesser-known taxa have opened up new opportunities for research into yeast biodiversity, functional genomics, and biotechnological innovation.

## 3. Focus on *Y. lipolytica*: A Biotechnological Model Organism

*Y. lipolytica* is increasingly recognized as a versatile, non-conventional yeast species with broad biotechnological applications. Originally noted for its ability to metabolize hydrophobic substrates, it has evolved into a prominent model organism for studying various cellular processes, including lipid metabolism, protein secretion, and morphological differentiation [32]. *Y. lipolytica* exhibits a unique genomic architecture that sets it apart from other ascomycetous yeasts. Notably, it has a high GC content, with coding sequences ranging between 57–59%, and a relatively large genome size of approximately 20.5 Mb, organized into six chromosomes. Unlike many yeasts, *Y. lipolytica* possesses an unusually high number of introns, with about 15% of its genes containing intronic sequences, compared to only 4% in *Saccharomyces cerevisiae*. Furthermore, *Y. lipolytica* exhibits a low genetic diversity among its strains, with a pan-genome comprising approximately 6528 genes, only slightly larger than its core genome of 6315 genes. This minimal variation suggests that *Y. lipolytica* is a recently evolving species with a relatively stable genome [33]. Biotechnological applications involving *Y. lipolytica* have been acknowledged as safe by the U.S. Food and Drug Administration (FDA), granting them GRAS (Generally Recognized as Safe) status in 2008. *Y. lipolytica* was granted Qualified Presumption of Safety (QPS) status by the European Food Safety Authority (EFSA) in 2018, officially recognizing it as a microorganism considered safe for use in food and feed production processes (Table 3 and Table 4). In Europe, the European Food Safety Authority (EFSA) has approved the yeast’s biomass, cultivated on waste-derived substrates, as a novel food ingredient for dietary supplements targeting individuals over the age of three. The recommended daily intake is limited to 3 grams for children aged 3–9 and 6 grams for older individuals. Regulation 2024/2044 further defines its permitted use across various food categories, such as nutritional meal replacements, clinical nutrition products, dairy analogues, baked goods, and soups [34].

In the EU, *Y. lipolytica* is typically used as a nutritional ingredient (protein/lipid source) or a gut flora stabilizer (probiotic). Its use is subject to EFSA’s safety evaluation depending on the strain, processing method, and target species. In the US, GRAS notices or AAFCO feed definitions are the most common pathways for commercialization. For example, dried *Y. lipolytica* biomass is recognized as a single-cell protein source for pets and livestock [34].

Given the growing body of scientific literature and evolving regulatory frameworks surrounding *Y. lipolytica*, it is clear that both the scientific community and food industry are increasingly interested in its potential applications [34].

**Table 3:** Overview of regulatory status, scope of use, and limitations related to the use of *Y. lipolytica* across different authorities in animal nutrition.

Authority	Regulatory Status	Scope of Use	Target Animals	Limitations/Conditions	Legal Basis/Source	Reference
FEFAC	Approved as feed material (2010)	Protein source, yeast biomass	Poultry, swine, ruminants	Biomass derived from fermentation using crude glycerol (non-GMO); compliance with EU feed hygiene standards; genetically modified micro-organisms shall be compliant with Regulation (EC) No 1829/2003 on genetically modified feed and food.	FEFAC Catalogue No. 00575-EN	[35]
EFSA (EU)	Authorized as feed material and probiotic additive (depending on strain and form)	Protein-rich biomass, lipid-rich biomass; used in feed for poultry, pigs, aquaculture (e.g., salmon, trout), and pets	Poultry, pigs, ruminants, fish, companion animals	Must comply with Directive 2002/32/EC (undesirable substances); must meet criteria of Regulation (EC) 767/2009 (placing on the market of feed)—but there is no direct reference to the biomass of *Yarrowia lipolytica*	EFSA FEEDAP Panel opinions; EU Catalogue of Feed Materials)	[34,36]
FDA (USA)	GRAS or approved feed ingredient (based on intended use) *	Could be used as a protein supplement, a source of lipids, or as a microbial additive	Poultry, swine, cattle, pets, aquaculture species	Product must comply with AAFCO definitions or have GRAS status for feed use	-	[34,37]

* *Yarrowia lipolytica* is not listed as an FDA-approved animal feed ingredient.

**Table 4:** Overview of regulatory status, scope of use, and limitations related to the use of *Y. lipolytica* across different authorities in human nutrition.

Authority	Regulatory Status	Scope of Use	Target Population	Limitations/Conditions	Legal Basis/Source	Reference
FDA (USA)	GRAS—Generally Recognized as Safe	Food biotechnology and industrial use: enzyme production, nutritional ingredients, additives	General population	No specific intake limits; safety based on toxicological data and historical use	GRAS Notice (e.g., GRN No. 000252 for *Y. lipolytica*)	[34,38]
EFSA (EU)	Novel Food—approved biomass	Dietary supplements, food for special medical purposes, meal replacements, dairy products, baked goods, soups	Individuals over 3 years of age	Max daily intake: 3 g for children (3–9 years), 6 g for older children and adults; biomass must be cultivated on specified waste-based media	Commission Implementing Regulation (EU) 2024/2044 of 6 March 2024—authorizing the placing on the market of *Y. lipolytica* biomass as a novel food	[34,39]

Recent studies have highlighted the successful metabolic engineering of *Y. lipolytica* to enhance lipid accumulation. By targeting key genes in lipid biosynthesis and β-oxidation pathways, researchers have increased the yeast’s capacity for producing lipids suitable for biofuel and oleochemical production [40].

*Y. lipolytica* is one of the earliest non-conventional yeast species explored for genetic manipulation. The first successful genetic modifications of this yeast were reported in the 1980s, following the development of molecular tools such as transformation protocols, shuttle vectors, and auxotrophic selection markers. These advances renewed interest in *Y. lipolytica* as a eukaryotic expression host for heterologous protein production. By the 2000s, the organism gained recognition as a robust platform for metabolic engineering and recombinant protein synthesis. This led to the commercialization of the YLEX™ expression system in 2006 and the establishment of proprietary expression platforms by companies such as Protéus, Oxyrane, and Yeastern Biotech. Further advances in synthetic biology allowed for the introduction of entire metabolic pathways, enabling the yeast to produce high-value compounds such as carotenoids, polyunsaturated fatty acids (PUFAs), aromatic molecules, citric acid, and human milk oligosaccharides (HMOs). In parallel, genetically engineered *Y. lipolytica* strains have also been developed for pharmaceutical applications, particularly for enzyme replacement therapies (ERTs) targeting metabolic and lysosomal disorders [5].

*Y. lipolytica* is an oleaginous yeast species known for its ability to accumulate intracellular lipids, exceeding 20% of its cell dry weight. The dominant components of these lipids are triacylglycerols (TAGs), which are rich in monounsaturated fatty acids—particularly oleic acid (C18:1) and linoleic acid (C18:2)—making them attractive for food, cosmetic, and biofuel applications. In addition to its lipid content, the biomass of *Y. lipolytica* contains approximately 50% protein (based on dry weight), with a favourable profile of essential amino acids, positioning it as a nutritionally valuable protein source. Furthermore, *Y. lipolytica* synthesizes mannoproteins that possess antioxidant properties and hold promise as natural stabilizers or functional additives in food processing [41].

The species is also a versatile producer of industrial enzymes, such as the following:

- Lipases, which catalyze fat hydrolysis and are valuable in cheese and dairy processing [42];
- Proteases, which break down proteins to improve flavour and texture in fermented foods [43,44];
- Esterases, which contribute to the biosynthesis of esters responsible for aroma development [45];

Another important trait is its ability to produce aromatic compounds like γ-decalactone, a lactone with a sweet, fruity fragrance used in the flavour and fragrance industries [9]. *Y. lipolytica* is commonly applied in beverage fermentation, where it enhances both the flavour profile and aromatic complexity of the final product. Moreover, it is considered a robust producer of citric acid, particularly when cultivated on agro-industrial waste streams. Its tolerance to low-pH and high-substrate concentrations further reinforces its industrial relevance, especially in sustainable bioprocessing strategies [9].

Advancements in synthetic biology have facilitated the development of novel genetic tools for *Y. lipolytica*. As discussed by Sanya and Onésime (2022) [46], innovations include modular gene expression systems, genome-editing techniques (e.g., CRISPR-Cas9), and the introduction of synthetic enzymes. These tools expand the organism’s potential for producing high-value compounds relevant to nutrition, pharmaceuticals, and environmental biotechnology [46].

*Y. lipolytica* is a strictly aerobic, oleaginous yeast capable of utilizing a wide range of carbon sources, including hydrophilic and hydrophobic substrates, for the biosynthesis of organic acids and lipids. It naturally inhabits lipid-rich environments such as dairy and poultry products or waste oils, and its adaptability also makes it a promising candidate for wastewater treatment [34,47,48,49,50].

This yeast synthesizes lipids through two main pathways: De Novo, using sugars and other hydrophilic carbon sources, and ex novo, utilizing hydrophobic substrates like oils and alkanes. In De Novo synthesis, excess carbon and nitrogen limitation stimulate the redirection of carbon flux toward acetyl-CoA and malonyl-CoA, leading to the formation of triacylglycerols (TAGs) [51]. However, it should be noted that hydrophobic substrates such as triacylglycerols or fatty acid esters are first hydrolysed by secreted or cell-bound lipases into free fatty acids, which are then selectively taken up and incorporated into stored TAGs or broken down by β-oxidation. Unsaturated fatty acids and C16:0 are preferentially assimilated, while C18:0 is poorly utilized due to negative selectivity [12].

*Y. lipolytica* is widely regarded as a model oleaginous yeast because of its ability to accumulate large amounts of lipids. Lipid storage occurs primarily in lipid bodies (LBs), where the majority of neutral lipids are present as triacylglycerols (TAGs) and smaller fractions as steryl esters (SEs). Under nutrient-limiting conditions, these stored lipids act as carbon reserves that can be mobilized by lipases such as Tgl3p and Tgl4p [52]. Although reports sometimes cite lipid accumulation exceeding 20%—and, under certain optimized conditions, even approach 70% of cell dry weight—extensive studies of wild strains demonstrate that, under standard nitrogen-limiting conditions, lipid accumulation typically ranges only from 4% to 19% of dry cell weight, with values above 20% being rare [53,54,55,56,57]. Consequently, lipid production in *Y. lipolytica* is highly strain-dependent and generally lower than is often assumed.

This yeast expresses multiple enzymes for the breakdown of hydrophobic substrates, including extracellular lipases and proteases. It can secrete biosurfactants, e.g., liposan, and produce emulsifying agents that improve substrate availability. Hydrocarbon uptake can also occur through direct contact with cell surface protrusions or hydrophobic invaginations, followed by translocation into the endoplasmic reticulum [52]. Alkanes are initially hydroxylated by cytochrome P450 monooxygenases, triggering conversion into alcohols and fatty acids. These enzymes are regulated by carbon source availability and localized to the ER membrane. Liposan synthesis, for example, is induced by growth on alkanes, but is suppressed by glucose [58,59]. In addition, *Y. lipolytica* possesses a unique fatty acid transport system that includes secreted binding proteins (eFbp1–4) and intracellular carriers such as YLSCP2, an SCP2 homologue responsible for shuttling fatty acids to target membranes. This distinguishes *Y. lipolytica* from *S. cerevisiae* and supports its specialization in the lipid metabolism [58,59,60].

Lastly, anabolic acyl-CoA synthetase activity has been confirmed in peroxisomes, mitochondria, and cytosol, playing a key role in incorporating exogenous fatty acids into cellular lipids. These traits collectively support the potential of *Y. lipolytica* as a platform organism for sustainable lipid-based bioproducts and green biotechnologies [58].

## 4. Species Diversity in the *Yarrowia* Genus

Species belonging to the genus *Yarrowia* are distinguished by their ability to undergo multilateral budding and the formation of pseudohyphae and true hyphae, which is called dimorphism. They produce asci through the conjugation of compatible mating types. While most *Yarrowia* species can share limited carbon compound assimilation and are physiologically similar, they can be differentiated based on sequences of the D1/D2 domains of the large subunit (LSU) rRNA gene and the internal transcribed spacer (ITS) regions [61].

### 4.1. Y. alimentaria

*Y. alimentaria* is a psychrotolerant species isolated from Brie Régalou cheese (CBS 12367), cured ham (CBS 10151), and a yoghurt production line (CBS 10149) [62]. It does not grow at 30 °C or in 10% NaCl, but shows variable growth at pH 3. Cells are ovoid to globose (3.0–6.0 × 4.0–7.0 µm), reproducing by multilateral budding and forming pseudohyphae and true hyphae on YM agar. This species has a specialized carbon and nitrogen assimilation profile, showing strong growth on D-ribose, erythritol, and D-glucono-1,5-lactone, but weak or no growth on polyols like D-mannitol, D-glucitol, and ribitol. No growth occurs on D-xylose, L-malic acid, or propane-1,2-diol, and utilization of L-sorbose, citrate, palatinose, uric acid, and gentiobiose is variable. Among nitrogen sources, ethylamine and D-proline may be utilized, while imidazole is weakly assimilated, and D-tryptophan is not used [63]. *Y. alimentaria* demonstrates high proteolytic activity (1.38 U/mL/min) and can effectively hydrolyze brewers’ spent grain proteins, accumulating significant levels of α-amino nitrogen [64]. Although it is less oleaginous than *Y. lipolytica*, it can accumulate up to 4.5% of its dry weight as lipids, with linoleic acid (C18:2 n-6) as the major fatty acid [62]. These features highlight its potential in food biotechnology and enzyme production.

### 4.2. Y. brassicae

*Y. brassicae* is a recently described yeast species within the *Yarrowia* genus, isolated from traditional Chinese pickled cabbage in Nanyang, Henan Province, China (strain CBS 15225T and CICC 33263T). Molecular analyses of the D1/D2 domain of the large subunit rRNA gene and ITS region revealed that it is phylogenetically distinct but closely related to *Y. divulgata*, with 2–7% sequence divergence. Phenotypic differentiation between *Y. brassicae* and *Y. divulgata* is limited, as both species share highly similar growth. *Y. brassicae* cells are ovoid with multilateral budding and occur singly or in pairs. Colonies on YM agar are white, smooth, and butyrous, and true hyphae and pseudohyphae are formed on corn meal agar. Notably, *Y. brassicae* does not ferment sugars, but can assimilate a limited range of carbon sources, including glucose, ethanol, glycerol, and citrate, while no growth occurs on sucrose, lactose, galactose, or methanol. It grows at 30 °C, but fails at 35 °C, tolerates 0.1% cycloheximide, but is sensitive to high salt (10% NaCl + 5% glucose) and acidic stress (1% acetic acid). Additionally, urease and diazonium blue B reactions are negative [61]. Despite its recent description, this species was not included in comparative genomic studies of telomerase RNA diversity across the *Yarrowia* clade [5]. Nevertheless, strains of *Y. brassicae* are available in major culture collections like CBS, expanding the genetic resources for studying non-conventional yeasts.

### 4.3. Y. bubula

*Y. bubula* is a psychrotrophic, anamorphic yeast species from bovine-derived substrates, including minced beef (CBS 12934) and pork (strain 441/4), with no observed ascospore formation [65]. This species exhibits distinct growth characteristics, with an inability to grow at 30 °C and an optimal range of 15–25 °C, making it cold-adapted [66]. It tolerates up to 10% NaCl and grows on unconventional substrates such as waste cooking oil (WCO), whey, and raw glycerol, highlighting its metabolic flexibility. Interestingly, strain NCIM 3590 lacks extracellular lipase activity in standard fermentations, correlating with low similarity (~42%) between its *LIP2* gene and that of *Y. lipolytica* [67]. However, isolates from dry-cured ham show significant lipolytic (HC > 2) and proteolytic activity in specific phases of meat processing, although they do not persist in later curing stages, likely due to salt stress and glucose limitations [68]. CBS 12934 also assimilates D-ribose, D-mannitol, and N-acetyl-D-glucosamine, differentiating it from *Y. yakushimensis* and *Candida alimentaria*. Lipid studies show NCIM 3590 accumulates up to 20% of its biomass as lipids, with earlier mobilization than *Y. lipolytica* (48 h vs. 72 h) and a higher proportion of saturated fatty acids (64.1% vs. 34.6%), indicating cold adaptation [66]. Overall, *Y. bubula* is a promising candidate for bioprocesses in aquaculture, waste valorization, and cold-environment applications.

### 4.4. Y. deformans

*Y. deformans* (strain YD22) is one of the new yeast species with potential biotechnological applications. *Y. deformans* YD22 can produce large amounts of a new extracellular thermostable alkaline protease. *Y. deformans* YD22 was isolated from fat-containing cheese, and several strains of *Y. deformans* (formerly known as *C. deformans*) have been reported to be lipolytic [69]. More unexpectedly, *Y. deformans* has been isolated from humans, particularly from the nail, which may raise concerns about its safety [5]. This species was originally described in 1934 by Zach as *Pseudomonilia deformans* and classified as *C. deformans* by Langeron and Guerra in 1938. In 1970, van Uden and Buckley recognized it as a synonym of *Candida* (*Yarrowia*) *lipolytica*. However, recent molecular studies based on the variability of nuclear rRNA gene sequences suggest that *C. deformans* is a distinct species with no known teleomorph associated with it. *C. deformans* strains obtained from South Africa can cross with strains present in the CBS yeast collection (Westerdijk Fungal Biodiversity Institute Collection) and produce the teleomorph *Yarrowia*, described as *Y. deformans* [70]. Research conducted by Bigey et al. (2003) based on sequences of the D1/D2 region of the large subunit (LSU) rRNA, Knutsen et al. (2007) using PCR analysis, DNA–DNA reassortment, and analysis of the ITS and LSU rRNA regions [71,72]. This species is heterothallic. The sacs are formed from conjugating yeast cells and are persistent, containing 1–2 (rarely 4) hat-shaped ascospores [70].

### 4.5. Y. divulgata

*Y. divulgata* (Latin divulgata—‘scattered’, referring to the wide geographical distribution of the new species) is a yeast that forms spherical, elongated, or ellipsoidal cells measuring 2.5–6 µm and 2.5–6.5 × 3.5–10 µm. They occur singly, in pairs, in short chains, or in small clusters. The optimum growth temperature is 25–30 °C. On MEA (2% malt extract agar) medium, it forms creamy, matt colonies ranging from almost smooth to wrinkled. Hyphae and pseudohyphae are observed in cultures. No ascospores are formed. The yeast does not show any fermentation capacity. *Y. divulgata* assimilates, among others, glucose, ribose, sorbitol, mannitol, glycerol, ethanol, gluconic acid, citrate, and hexadecane, as well as some aromatic compounds and polyhydric alcohols. It does not assimilate most disaccharides, methanol, xylitol, or nitrogen compounds such as nitrates and creatine. This species requires the presence of vitamins in the medium [73]. *Y. divulgata* has a very similar growth profile to *Y. brassicae* and is phenotypically difficult to distinguish. The differences include, among others, the ability to assimilate inulin and xylitol. To clearly distinguish between these species, it is recommended to analyze the sequence of the D1/D2 LSU rRNA gene and ITS regions [61].

### 4.6. Y. galli

*Yarrowia (Candida) galli* yeast was first described from chicken tissue (breast and liver) in 2004 and has been sporadically isolated in clinical settings. It is considered a rare clinical fungal pathogen capable of causing superficial infections in humans. The first clinical strain was isolated from the nail of a woman with mycosis [74]. Yeast cells are spherical, ellipsoidal, or elongated in shape, measuring 2–4 × 1.5–6.7 (−12) μm, produced singly, in pairs, or in small clusters, with multilateral budding, forming pseudohyphae and hyphae 1–1.5 μm wide. It exhibits growth at high glucose concentrations (up to 60%). It is distinguished by a stable phenotype, with minor differences compared to previously described strains. Identification based on the ITS rDNA region sequence confirmed its species affiliation [75]. The species is closely related to the oleaginous yeast *Y. lipolytica*. In addition, it exhibits significant morphological diversity. In cultures on YPD medium at 25 °C, several colony forms were observed: yeast, fluffy, sticky, and compact. Each contained yeast cells and hyphae, and the identity of these morphotypes as *Y. galli* was confirmed by sequencing. Genetic and phenotypic data suggest that *Y. galli* may be an opportunistic fungal pathogen for humans [74].

### 4.7. Y. hollandica

*Y. hollandica* was first described as *Candida hollandica* from the back of a cow in the Netherlands and was later reassigned to the *Yarrowia* clade based on rDNA sequence analysis. The type strain CBS 4855 is the reference for this species. *Y. hollandica* shows broad, but variable carbon source utilization, including growth on D-galactose, ribitol, D-glucitol, D-mannitol, D-glucono-1,5-lactone, citrate, propane-1,2-diol, uric acid, and gentiobiose. Growth on L-sorbose, D-ribose, D-xylose, erythritol, palatinose, and L-malic acid is variable, and all strains grow well on D-glucose, glycerol, D-gluconate, DL-lactate, succinate, and galactaric acid [71]. It also utilizes ethylamine, imidazole, and D-proline as nitrogen sources. This species grows at 30 °C, tolerates 10% NaCl, and can grow at acidic pH. It has been identified as a moderate protease producer, with strains D4 and D11 showing potential for protein hydrolysis in dry-aged beef fermentation [76]. Its proteolytic potential and moderate halotolerance make it an attractive non-conventional yeast for biotechnological applications.

### 4.8. Y. keelungensis

*Y. keelungensis* fa (kee.lung.en’sis. NL nom. masc. adj.) refers to the city of Keelung in Taiwan, near the place where the organism was first isolated. This strain exhibits high cell surface hydrophobicity (>90%) and moderate resistance to UV radiation. Phylogenetically, it is most closely related to *C. osloensis*, but there are differences in the rDNA D1/D2 sequence (3.7%), which confirms their species distinctiveness. It does not form ascospores. Therefore, it was considered an anamorph and described as a new species, *Y. keelungensis*. The cells of this yeast are ellipsoidal to elongated, 3.0–7.5 μm long and 2.5–4.0 μm wide, and occur singly, in pairs, or groups. The colonies are smooth, with a complete margin, dull, and brownish-white. Fermentation does not occur. Growth occurs at temperatures between 25 °C and 35 °C [77]. These yeasts are capable of accumulating lipids in amounts ≥ 20% dry weight (22.2% DW) and are efficient producers of xylitol, achieving a yield of 0.75 g/g xylose consumed and a titer of 19.0 g/L, using xylose as the primary substrate after depleting available glucose [78].

### 4.9. Y. osloensis

It is described as *Candida osloensis*, but belongs to the *Yarrowia* clade [56]. The cells are ovoid to spherical, measuring 3.0–6.0 × 3.0–9.0 μm, occur singly, in pairs, and in small clusters, and reproduce by multiple budding. The optimal growth temperature is 25 °C. The species was isolated from yoghurt [77]. It has been reported that *C. oslonensis* utilizes galactose and sorbose as carbon sources, which are poorly metabolized, if at all, by other species. *Y. osloensis* stands out as one of the best mannitol producers within the *Yarrowia* genus, apart from the species *Y. lipolytica*, where some strains can produce almost exclusively mannitol within high final concentration optimization, with high conversion efficiency after fermentation optimization [79,80]. Additionally, *Y. osloensis* also produced significant amounts of mannitol from glucose and fructose, making it a versatile representative of the genus in terms of the utilization of different carbon sources [79].

### 4.10. Y. parophonii

*Y. parophonii* was isolated from the gut of the ground beetle *Parophonus hirsutulus* in Bulgaria. ITS and D1/D2 rDNA analyses of seven strains (CBS 12427–12471) revealed high intra-species similarity and separation from its closest relative, *Y. oslonensis* (16 substitutions in LSU rDNA), although minor ITS differences (one substitution and one gap) indicate the need for further phylogenetic study [81,82]. This species assimilates N-acetyl-D-glucosamine, D-mannitol, and D-galactose, and can grow in vitamin-free media. On GPYA and MA at 25 °C, colonies are cream, smooth, convex, and produce hyphae; after 1–2 weeks on YM agar, hat-shaped ascospores may form. *Y. parophonii* exhibits moderate proteolytic activity (0.80 U/mL/min), higher than *Y. bubula* or *Y. brassicae*, but lower than *Y. galli* and *Y. lipolytica* [64]. Its alkaline protease (~65 kDa) shares 26% similarity with those from *Yarrowia* sp. strains E02 and B02, indicating potential biotechnological value. Notably, protease activity in *Y. paraphonii, Y. alimentaria*, and *Y. lipolytica* remains stable over time, suggesting constitutive secretion. Its ability to grow without external vitamins and its distinct assimilation profile highlight its potential as an insect symbiont and as a source of biotechnologically relevant enzymes [68].

### 4.11. Y. phangngensis

The name *Y. phangngensis* refers to the province of Phang-Nga in Thailand, where the first two strains were isolated. Yeast cells are spherical, ellipsoidal to elongated (1.5–4 × 2–7 μm), occur singly or in pairs, and bud multilaterally. On YM agar, they form creamy, hairy, raised colonies with a fibrous edge. In cultures, they produce hyphae and pseudohyphae, but do not form ascospores. They do not ferment sugars. They tolerate high glucose concentrations and assimilate, among others, glycerol, erythritol, mannitol, gluconic and lactic acid, and ethanol, but do not assimilate most disaccharides, nitrates, or inulin. Growth is possible up to a temperature of 37 °C, but requires the presence of vitamins [79,83]. Sequencing of the mitochondrial DNA of *C. phangngensis* confirmed its affiliation with the *Yarrowia* clade [5]. *Y. pangngensis* is distinguished by a specific fatty acid profile compared to other species of the genus, especially *Y. lipolytica*. Strains of this species produce a significantly higher proportion of 16-carbon fatty acids, mainly palmitic acid (C16:0) and palmitoleic acid (C16:1), accounting for approximately 35% of total fatty acids, while in *Y. lipolytica*, this is only 18%. The maximum lipid content in *Y. phangngensis* was up to four times higher than that of the strain, *Y. lipolytica* W29, making it one of the best lipid producers [78].

### 4.12. Y. porcina

*Y. porcina* was originally isolated from minced pork in Hungary (CBS 12935) and from subsurface water in the Rio Doce River, Brazil (CBS 12932) [65]. It is among the few heterothallic teleomorphic species of the genus, producing sexual structures only when compatible mating types are combined. It can grow at 25–30 °C. The asci contain ellipsoidal, allantoid, or cap-shaped ascospores embedded in distinctive areolar material—an uncommon feature among yeasts—and measure 3.5–6.0 × 9.5–17.0 µm. Interestingly, budding can occur from mature ascospores after several months, confirming their developmental potential. Phenotypically, *Y. porcina* is challenging to distinguish from related species like *Y. deformans, Y. divulgata, Y. keelungensis*, and *Y. lipolytica*, which complicates traditional identification methods [84]. This challenge was evident in studies where 26% of meat isolates could not be conclusively identified by phenotype alone. However, molecular fingerprinting and D1/D2 rRNA sequencing resolved its taxonomic status, placing it in a group of novel species alongside *Y. bubula* and *Y. divulgata*. Notably, unlike *Y. lipolytica*, *Y. porcina* does not produce extracellular lipase under standard conditions [68]. The combination of its unique reproductive traits and diverse ecological distribution underscores its importance in understanding biodiversity and evolution within the *Yarrowia* genus.

### 4.13. Y. yakushimensis

Yeast cells of *Yarrowia yakushimensis* fa (the species name refers to the origin of the isolates, Yakushima Island in Kagoshima Prefecture, Japan; fa—asexual form) are ovoid to elongated (2.5–9.5 × 4.5–12.0 μm), occur singly, in pairs, or clusters, and reproduce by multilateral budding. It forms pseudohyphae, but no ascospores. On GPY agar medium, colonies are buttery and creamy, with slightly wavy edges. Growth occurs at 25 °C, but not at 30 °C. The species is distinguished by its ability to assimilate D-mannitol and its lack of growth at 30 °C [70]. Based on the sequence of the D1/D2 region of the LSU, the rRNA subunit of *Y. yakushimensis* forms the closest phylogenetic clade with *Y. lipolytica* [5]. Isolated from the intestines of insects [67]. In glucose cultures with yeast extract, the lipid content gradually decreased over time from 6% to 4.7% of dry cell weight, and the main accumulated fatty acid is linoleic acid (C18:2 n-6), which distinguishes it from most species of the genus *Yarrowia* [62]. This lipid profile makes *Y. yakushimensis* potentially interesting for biotechnological applications that require the production of polyunsaturated fatty acids.

### 4.14. Physiological and Genomic Overview of Yarrowia Species

The main physiological and metabolic characteristics of species within the genus *Yarrowia* are summarized in Table 5. Across most species, detailed quantitative data on salt tolerance are limited; however, 10% NaCl tolerance appears to be a common threshold for salt sensitivity or tolerance among several species. Optimal growth temperature is generally around 25 °C, although some species exhibit broader or narrower temperature ranges—for example, *Y. bubula* shows psychrophilic traits with optimal growth between 15 and 25 °C, whereas *Y. phangngensis* can grow up to 37 °C, indicating thermal adaptability within the genus.

Metabolic traits vary widely, with some species showing strong lipolytic or proteolytic activities. Notably, *Y. bubula* demonstrates pronounced lipolytic activity relevant for meat processing applications, whereas *Y. deformans* produces thermostable and alkaline proteases, highlighting its potential for industrial enzyme production. In contrast, *Y. brassicae* and *Y. divulgata* lack urease activity, requiring molecular methods for precise differentiation due to their phenotypic similarity.

Some *Yarrowia* species exhibit an ability to produce polyols, e.g., *Y. keelungensis* efficiently produces xylitol [78], and *Y. osloensis* produces mannitol in significant amounts [79], while *Y. alimentaria* shows poor growth on polyols. Species of this genus can also assimilate and produce organic acids (e.g., gluconic acid, gluconic acid-1,5-lactone, citric acid, succinic acid), but these are not currently commercially exploited [63]. In addition, species such as *Y. phangngensis*, *Y. yakushimensis*, and *Y. bubula* have signficiant potential for the production of lipids and enzymes, which may support the food and biotechnology industries.

Lipid accumulation is generally underreported, but presents interesting species-specific profiles (Table 5). For instance, *Y. phangngensis* shows high lipid production—up to four times that of the model *Y. lipolytica*—with a distinctive fatty acid composition rich in C16:0 and C16:1. *Y. keelungensis* also accumulates lipids exceeding 20% dry weight, coupled with high xylitol yields, suggesting dual biotechnological potential. Conversely, *Y. alimentaria* accumulates relatively low lipids (~4.5% dry weight), but is notable for its strong protease activity and tolerance to low pH, features valuable for protein hydrolysis in acidic conditions. Additionally, *Y. yakushimensis* exhibits a PUFA-rich lipid profile, dominated by linoleic acid, which offers potential for nutritional biotechnology.

**Table 5:** The most important comparative features of the described species.

Species	Growth Temperature	Salt Tolerance	Metabolic Traits	Lipid Accumulation	Other Key Traits	Reference
*Y. alimentaria*	Not at 30 °C	Sensitive (10% NaCl)	High protease (1.38 U/mL/min)	~ 4.5% dry weight (low)	pH 3-tolerant, strong brewers’ grain-protein hydrolysis	[62,63,64]
*Y. brassicae*	up to 30 °C, fails at 35 °C	Sensitive (10% NaCl)	Urease and diazonium blue B reactions are negative, no fermentation	Not reported	Close to *Y. divulgata*, difficult to distinguish without molecular analysis	[5,61]
*Y. bubula*	15–25 °C (psychrofilic)	Tolerates 10% NaCl	Strong lipolytic activity (meat processing phase); strain-dependent protease production	Up to 20%, high SFA	Grown on WCO, glycerol, and whey	[65,66,67,68]
*Y. deformans*	25 °C	Not reported	High thermostable, alkaline protease, lipolytic	Lipolytic species	Teleomorphic, potential clinical relevance	[5,69,70,71,72]
*Y. divulgata*	25–30 °C	Not reported	Non-fermentative	Not reported	Vitamin-requiring, phenotypically very similar to *Y. brassicae*	[61,73]
*Y. galli*	25 °C, unable to grow at 37 °C	Not reported	Variable morphology, possible clinical significance	Not reported	A rare human pathogen may cause superficial infections	[74,75]
*Y. hollandica*	Grows at 30 °C	Tolerates 10% NaCl	Moderate protease production	Not reported	Variable carbon source utilization, acid tolerance, and useful in dry-aged beef processes	[71,76]
*Y. keelungensis*	25–35 °C	Not reported	Non—fermentative, high cell surface, hydrophobicity, xylitol yield	≥20% DW, xylitol production	UV-resistant, anamorphic	[77,78]
*Y. osloensis*	Optimal at 25 °C	Not reported	Excellent mannitol producer (0.54 g/g substrate)	Not reported	One of the top mannitol-producing species in the genus	[56,71,79,80]
*Y. parophonii*	Up to 25 °C	Not reported	Moderate protease (0.80 U/mL/min), enzyme biotech potential	Not reported	Vitamin–free growth, hyphae, hat–shaped ascospores;	[64,68,81,82]
*Y. phangngensis*	Up to 37 °C	Not reported	High—lipid production (up to twofold higher than *Y. lipolytica*)	High in C16:0 and C16:1	Vitamin requirement, distinct fatty acid profile	[5,78,79,83]
*Y. porcina*	At 25–30 °C	10% NaCl	No extracellular lipase, teleomorphic	Not reported	Teleomorphic, asci with ascospores, heterothallic	[65,68,84]
*Y. yakushimensis*	25 °C, not at 30 °C	Not reported	Non-fermentative, lipid content decreases over time	From 6% to 4.7% DW	Linoleic acid dominant, PUFA-focused biotech potential	[5,62,67,70]

Species within the genus *Yarrowia* are primarily classified based on phylogenetic analyses of ribosomal DNA sequences, supported by phenotypic and physiological traits. The most widely used molecular markers are the D1/D2 region, which refers to a variable segment at the 5′ end of the large subunit (LSU, 28S) ribosomal RNA (rRNA) gene and the internal transcribed spacer (ITS) region, which is a non-coding DNA region located between the rRNA genes (18S, 5.8S, and 28S) [85]. For yeasts in general, including *Yarrowia*, sequence divergence in the D1/D2 LSU rRNA domain of ≤1% (≈0–3 nucleotide differences out of ~600 bp) is typically considered to indicate conspecific strains, while differences greater than this threshold suggest distinct species [86,87]. For ITS regions, which evolve more rapidly, intraspecific variability is usually less than 1–2%, whereas interspecific divergence often exceeds 3–5%. These cutoffs are consistent with phylogenetic studies of *Yarrowia*, where species such as *Y. brassicae* and *Y. divulgata* show less than 3% ITS divergence from *Y. lipolytica*, but very low intraspecific variation among their own strains [88].

In addition to molecular data, secondary criteria include differences in physiology (e.g., temperature and salt tolerance, lipid metabolism, urease, and DBB tests—diazonium blue B test) and ecological or geographic separation. These phenotypic characteristics are particularly important for distinguishing very closely related species or when sequence similarity is near the cutoff thresholds. For example, *Y. brassicae* and *Y. divulgata* are DBB-negative species, in contrast to some other members of this genus. The DBB test result was one of the phenotypic traits used to confirm their distinct species status, alongside D1/D2 and ITS rRNA sequence analyses and additional physiological tests [88].

Table 6 summarizes genomic data available in the NCBI database for yeast species of the genus *Yarrowia*. For each species, all available genome assemblies are aggregated, with numerical values presented as ranges where applicable. The number of scaffolds or chromosomes per assembly varies widely among species, ranging from as few as 6 scaffolds or chromosomes in *Y. lipolytica* reference strains and some contig-level assemblies to up to 882 scaffolds in *Y. alimentaria*. This variation reflects differences in assembly completeness and sequencing technology used. The table also highlights that several species possess multiple genome assemblies in NCBI, with *Y. lipolytica* having the most extensive genomic representation (48 assemblies), while others have only one or two. This discrepancy underscores the focus on *Y. lipolytica* as a model organism in this genus.

Genome sizes across the genus also show notable variability, spanning from approximately 16.2 Mb in *Y. phangngaensis* to over 30 Mb in *Y. porcina*, with most species falling within the 19–25 Mb range (Table 6). The GC content is relatively conserved, generally ranging between 43.5% and 51%, suggesting a stable genomic base composition within the genus. The number of predicted protein-coding genes (CDS) is reported for a subset of assemblies, predominantly for *Y. lipolytica* strains and some *Yarrowia* sp. isolates, on average. *Yarrowia* species possess around 7000 protein-coding genes, with *Y. lipolytica* displaying the highest gene counts, from approximately 7000 to 8700 genes, consistent with its relatively well-annotated reference genomes. For many species, this information is unavailable or not reported in the database.

Three reference strains of *Y. lipolytica*—CLIB122, PO1f, and WSH-Z06—are treated separately due to their high-quality chromosome-level assemblies and their importance as standard genomic resources. *Y. lipolytica* CLIB122, which was sequenced by the French National Institute for Agricultural Research (INRA), is a widely used reference strain notable for its well-annotated genome and utility in industrial biotechnology [89]. *Y. lipolytica* PO1f was sequenced by the University of Delaware and is a genetically tractable laboratory strain commonly used in molecular biology studies [90]. Finally, *Y. lipolytica* WSH-Z06 was sequenced by the Chinese Academy of Sciences and is distinguished by its complete genome assembly and potential for studies in lipid metabolism [91].

Overall, this comparative genomic overview provides a valuable foundation for understanding the genetic diversity and genomic architecture within the *Yarrowia* genus, which can guide future functional studies and biotechnological applications. While the genome of *Y. lipolytica* is well characterized and serves as a model for research and biotechnological applications, the genomes of other *Yarrowia* species remain less explored. Further genomic studies on these species may reveal novel biological features and expand opportunities for genetic engineering and industrial use in the future.

## 5. Conclusions

Following a comprehensive review and analysis of each strain of the *Yarrowia* species, it was determined that these species exhibit substantial morphological and metabolic similarities, but divergent characteristics in terms of genomics, adaptability, and application potential. *Y. lipolytica* is currently the most prominent and extensively utilized biotechnological model; nevertheless, there is mounting interest in other species with distinctive properties, as evidenced by the annual surge in the number of publications and scientific studies, as well as the development of genetic tools, indicating the dynamic development of this field of science. Increasingly precise legal regulations are conducive to the safe introduction of *Yarrowia* products into food, feed, and pharmaceuticals. However, it should be noted that the amount of available data is limited, resulting in a lack of understanding of some aspects of the characteristics of these species. Hence, the focus of this article is on *Y. lipolytica* as the most versatile and commercially exploited species, and its applications are highlighted.

In order to comprehend the preliminary characteristics of new species, further comparative studies are required, particularly by employing modern genomic and metabolomic techniques. The genus *Yarrowia* displays considerable physiological and metabolic diversity, with several species possessing unique traits that warrant further investigation. Expanding research on salt tolerance, lipolytic and proteolytic capacities, and lipid biosynthesis could unlock novel applications in food, pharmaceutical, and industrial biotechnology. This will allow for the full exploitation of the biotechnological potential of these newly discovered yeasts. Nevertheless, such a review provides a solid foundation for analysis.

## Figures and Tables

**Table 1:** Taxonomy of the *Yarrowia* yeast [own elaboration based on [1]].

Kingdom	*Funghi*
Subkingdom	*Dikaryota*
Phylum	*Ascomycota*
Subphylum	*Saccharomyconita*
Class	*Saccharomycetes*
Order	*Saccharomycetales*
Family	*Dipodascaceae*
Genus	*Yarrowia*

**Table 6:** Comparative genomic overview of *Yarrowia* species. All data were collected from the NCBI database at 10 July 2025. Species—Full species name; Assembly accession—NCBI accession ID for the genome assembly; Year of release—Year the genome was released or updated in NCBI; Assembly level—Assembly status: Chromosome, Scaffold, Contig; No. of scaffolds/chromosomes—Number of scaffolds or chromosomes in the assembly; Genome size (Mb)—Total genome length in megabases; GC content (%)—Proportion of guanine-cytosine pairs in genome; No. of genes (CDS)—Number of protein-coding genes (CDS = coding DNA sequences); No. of genome assemblies (NCBI)—Total number of genome assemblies for that species in NCBI Genomes; Source/Institution—Origin of the sequencing data or research group.

Species	Year	Assembly Level	No. of Scaffolds/ Chromosomes	Genome Size (Mb)	GC Content (%)	No. of Genes (CDS)	No. of Assemblies (NCBI)
*Y. alimentaria*	2018–2023	Scaffold	11–882	19.48–19.83	49.50–49.50	-	2
*Y. brassicae (nom. inval.)*	2023	Scaffold	407–407	20.95	50.50–50.50	-	1
*Y. bubula*	2018–2023	Scaffold	28–811	20.21–20.90	47.00–47.00	-	3
*Y. deformans*	2016–2023	Scaffold	42–426	20.55–21.12	50.00–50.50	-	3
*Y. divulgata (nom. inval.)*	2018	Scaffold	19–488	21.24–21.44	50.00–50.50	-	2
*Yarrowia galli*	2018–2023	Scaffold	6–482	22.68–23.02	49.50–49.50	-	3
*Y. hollandica*	2018–2023	Scaffold	18–371	19.50–20.07	48.00–48.00	-	2
*Y. keelungensis*	2016–2023	Scaffold	39–474	21.64–21.82	48.00–48.50	-	3
*Y. lipolytica*	2015–2025	Chromosome, Complete Genome, Contig, Scaffold	6–530	0.01–21.25	47.00–50.50	7082–8746	48
*Y. lipolytica CLIB122* *	2004	Chromosome	6–6	20.50	49.00–49.00	7144–7334	2
*Y. lipolytica PO1f* *	2014–2024	Chromosome, Complete Genome	6–6	20.51–20.62	49.00–49.00	-	2
*Y. lipolytica WSH-Z06* *	2014	Complete Genome	6–6	20.09	49.00–49.00	-	1
*Y. osloensis*	2018–2023	Scaffold	37–536	22.97–23.25	51.00–51.00	-	2
*Y. phangngaensis*	2018–2023	Scaffold	9–435	16.21–16.23	43.50–43.50	-	2
*Y. porcina*	2018–2023	Scaffold	43–887	30.00–30.43	44.50–44.50	-	2
*Yarrowia* sp. *B02*	2021	Contig	6–6	19.98	51.00–51.00	7047–7047	1
*Yarrowia* sp. *C11*	2021	Contig	11–11	24.88	46.50–46.50	6900–6900	1
*Yarrowia* sp. *E02*	2021	Contig	9–9	25.05	46.50–46.50	6879–6879	1
*Yarrowia* sp. *JCM 30694*	2016	Scaffold	19–19	21.76	46.50–46.50	-	1
*Yarrowia* sp. *JCM 30695*	2016	Scaffold	41–41	21.90	48.50–48.50	-	1
*Yarrowia* sp. *JCM 30696*	2016	Scaffold	39–39	21.75	48.50–48.50	-	1
*Y. yakushimensis*	2018–2023	Scaffold	7–218	18.43–18.84	48.00–48.50	-	2

* Three reference strains of *Yarrowia lipolytica*—CLIB122, PO1f, and WSH-Z06—were excluded from the aggregated row for this species due to their reference status and the availability of complete, high-quality chromosome-level genome assemblies. These strains are considered separately as representative genomic resources.

## Data Availability

No new data were created or analyzed in this study.
